# Body contouring after bariatric surgeries in Jordan: Awareness, prevalence, and challenges: A multicentric cross-sectional study

**DOI:** 10.1097/MD.0000000000034279

**Published:** 2023-08-18

**Authors:** Marzouq N. Amarin, Amani A. Atallah, Mohammad Z.A. Rashdan, Izdiad A. Atallah, Majdi M. Khrais, Yazan H. Jaber, Afnan A. Atallah, Omar M. Ismail, Kamel A. Jaber, Taima K. Fkheideh, Raed N. Altaher

**Affiliations:** a Department of General Surgery, School of Medicine, The University of Jordan, Amman, Jordan; b Department of Educational Leadership and Foundations, School of Educational Sciences, The University of Jordan, Amman, Jordan; c School of Medicine, The University of Jordan, Amman, Jordan.

**Keywords:** bariatric surgery, body contouring, obesity

## Abstract

Body-contouring surgeries are known to improve the quality of life and body image of patients undergoing bariatric surgery. However, only a small number of patients choose to undergo body-contouring surgeries. This study evaluated the prevalence of body-contouring surgeries among patients who underwent bariatric surgery in Jordan, and identified the limitations encountered. This study is an observational multicentric cross-sectional study. A validated questionnaire was distributed to patients who had undergone bariatric surgeries between July 2017 and June 2021 at the University of Jordan Hospital and a bariatric surgery private center in Amman, Jordan. Inclusion criteria were based on the type of bariatric surgery (Sleeve Gastrectomy or Roux-En-Y gastric bypass) and the surgery date falling within the study period, with participation requiring the completion of an online questionnaire. Collected data was analyzed using various statistical tests, with a predetermined alpha level of 0.05 to determine statistical significance. Of 451 eligible participants, a total of 305 patients completed the validated questionnaire. Of these, 11 responses were excluded due to incomplete data. The analysis focused on remaining 294 participants who underwent bariatric surgery between July 2017 and June 2021, with only 7 participants (2.4%) electing to undergo body-contouring surgeries. The primary barriers to body-contouring surgeries reported by participants were cost (62.7%) and fear of postoperative complications (31.8%). Females exhibited a significantly greater desire for body-contouring surgeries (*P* = .000), which was also related to the percentage of total weight loss following bariatric surgery (*P* = .025). However, no significant associations were observed between desiring body-contouring surgeries and marital status (*P* = .734) or employment status (*P* = .319). The low rate of body-contouring surgeries in Jordan reflects the importance of strengthening the patient-physician relationship through targeted education efforts that emphasize the expected consequences of bariatric surgery and the available solutions to address them. Additionally, encouraging collaboration among caregivers, healthcare authorities, and insurance providers is necessary to develop more inclusive treatment plans that are tailored to meet the diverse needs and socioeconomic backgrounds of patients.

## 1. Introduction

Obesity rates have tripled since 1975, and this is not only an issue for adults. Recent statistics show that more than 30 to 300 million children and adolescents worldwide are considered overweight or obese.^[1]^

Despite diverse recommendations for lifestyle modifications, diet regimens, and consistent physical activity, evidence-based medical and surgical interventions are essential in the management of obesity. This has led to a rapid growth in bariatric surgery (BS) worldwide.^[2]^ Bariatric surgeries have indications, complications, and effects on patients’ quality of life, which subsequently led to a dramatic increase in the need for effective body-contouring surgeries (BCSs) after weight loss, which is desired by more than 60% to 80% of patients after BS.^[3,4]^ However, desire alone is not sufficient to motivate patients to undergo BCSs. The rates of BCSs after BS vary in the literature, with most studies reporting rates of 11% to 14%.^[4–6]^ This study aimed to describe Jordanians’ awareness and knowledge of BCSs and estimate their prevalence. Additionally, we aimed to identify the limitations reported by our population and compare them with those reported in the literature.

## 2. Materials and Methods

**Figure 1.**
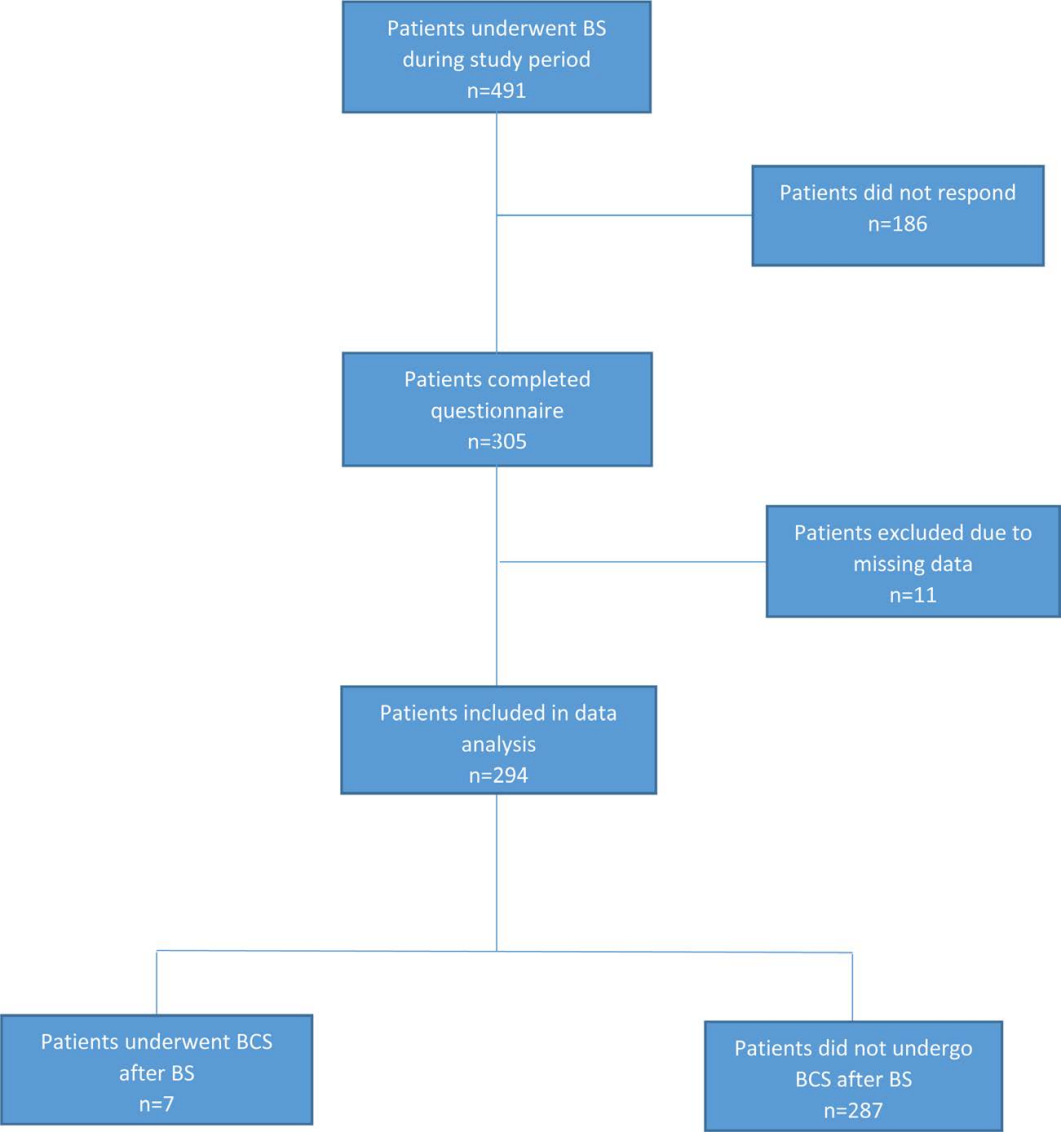
Flow chart of participants included in the study. BCS = body-contouring surgery, BS = bariatric surgery.

Jordan University Hospital (JUH) is one of the largest tertiary hospitals in Amman, the capital of Jordan. The bariatric surgery (BS) department at the JUH has experienced significant growth in recent years. From July 2017 to June 2021, 351 patients underwent BSs at the JUH. After obtaining the study approval of the Institutional Review Board at the University of Jordan Hospital, we aimed to reach out to all 351 patients as well as an additional 100 patients who underwent BS at one of the largest private medical facilities in Amman between January and June of 2021. Including participants from a university hospital as well as a private center aimed to minimize any potential selection bias. We distributed a validated questionnaire to all patients. Of the 451 patients, 305 completed the questionnaire (response rate: 67.6%). The inclusion criteria were BS type (sleeve gastrectomy or Roux-en-Y bypass), surgery date falling within the study period, and completion of the questionnaire. Responses that did not meet these criteria or contained duplicates were excluded (n = 11). The remaining 294 responses were collected and entered into a database for the analysis (Minitab 16). The participants were divided into 2 groups: group (A) included 287 respondents who did not undergo any body-contouring surgery (BCS) after weight loss, and group (B) which included 7 respondents who underwent BCSs after BSs. Three main variables were assessed in each group in addition to multiple other observations with a prospectively determined alpha level of.05 to indicate significant differences. Figure 1 shows the flow chart of participants in the study. The main variables were sex, marital status, and employment status. Various statistical tests were used based on the data and hypotheses of the study, including the Analysis of Variance test to determine the differences in continuous responses based on the levels of the study variables, and the Chi-square test to analyze yes or no type questions in group A in relation to the main 3 variables of the study. The two-sample *t* test was used to determine if there was a significant difference in total weight loss% after the 2 types of studied BS and its impact on desiring subsequent BCS. The Fisher’s exact test was specifically used to analyze the responses of group (B) instead of the Chi-square test to obtain more accurate results due to the small sample size.

## 3. Results

Of 451 potentially eligible participants, a total of 305 patients completed the validated questionnaire. Of these, 11 responses were excluded due to incomplete data. The analysis focused on the remaining 294 participants who underwent bariatric surgery between July 2017 and June 2021. Of the 294 included participants, 215 females made up the majority (73.1%), compared to 79 males (26.8%). Sixty-three percent (63%) of the participants were married, and 33% were single. The remaining 4% were either divorced or widowed. Of all participants, only 44.8% were formally employed. Laparoscopic sleeve gastrectomy (LSG) was performed in 232 participants (78.9%), while 70 participants (23.8%) underwent laparoscopic Roux-en-Y gastric bypass (RYGB). Only 7 (2.4%) participants underwent body-contouring surgeries (BCSs) after bariatric surgery (BS). Data from participants who did not undergo BCSs (Group A: 97.6%) were analyzed separately from those who underwent BCSs (Group B: 2.4%).

There was no statistically significant difference between the sex of the participants and the type of BS performed (*P* = .953), as shown in Table 1. Females were significantly more willing to undergo BCS than males; 76.9% of females desired one or more BCS (*P* = .000).

**Table 1:** Relationship between sex and observations in group A.

Questions	Sex variable		*P* value
	Male	Female	
Type of BS			
Sleeve gastrectomy	78.2%	77.9%	.953
Roux-en-Y Bypass	21.8%	22.1%	
Desire to perform BCS			
Yes	52.6%	76.9%	.000
No	47.4%	23.1%	
Adequate knowledge of BCS			
Yes	59%	60.8%	.774
No	41%	39.2%	
Surgeon’s help in making decision			
Yes	53.8%	46.5%	.268
No	46.2%	53.5%	
Expect to improve performance of daily activity			
Yes	69.2%	74.8%	.398
No	30.8%	25.2%	
Expect to improve social relationships			
Yes	67.9%	66.8%	.856
No	32.1%	33.2%	
Expect to improve self-confidence			
Yes	70.5%	79.7%	.096
No	29.5%	20.3%	

BCS = body-contouring surgery, BS = bariatric surgery.

Married participants were more likely to undergo LSG (76.6%) than RYGB (23.4%) (*P* = .040). This significance was not observed among single, widowed, or divorced participants.

Seventy-two percent (72%) of the participants desired one or more BCS, which was not related to their marital status (*P* = .734). Marital status did not affect participants’ knowledge of BCSs as well (*P* = .578).

Employment status was not significantly related to the type of BS performed, desire to perform BCS after weight loss, or degree of knowledge about BCSs (*P* = .256,.319, and.623, respectively). However, employed participants were more inclined to believe that performing BCS would improve their social relationships (72.8%) than unemployed participants (62.3%) (*P* = .055).

Participants with a higher baseline Body Mass Index (BMI) with a mean BMI of 45.9 kg/m^2^ were more likely to undergo LSG than those with a mean BMI of 42.8 kg/m^2^ in participants who underwent RYGB (*P* = .045). The analysis also showed a statistically significant difference in weight loss after LSG compared to RYGB; the mean total weight loss percentage (TWL%) after LSG was 34.3%, compared to 29.0% after RYGB (*P* = .000).

There was a statistically significant relationship between TWL% and desire to perform BCSs (*P* = .025). However, there was no statistically significant relationship between the type of BS performed and desire to perform BCS (*P* = .755).

Participants who underwent LSG had stable weight approximately 15 months after surgery, compared to weight stabilization 13 months after RYGB (*P* = .258).

A total of 287 participants reported 367 different sources of knowledge of BCSs. The most common sources of knowledge were social media and magazines, reported by 150 participants (50.9%), followed by experiences of relatives and friends, reported by 117 participants (38.3%), and less commonly, from a specialist surgeon, reported by only 100 participants (34.5%).

The main barriers and limitations preventing participants from proceeding with the desired BCSs were reported by 201 participants. Unaffordable cost was the most common barrier reported by 126 participants (62.7%), followed by fear of complications and insufficient knowledge of options, reported by 64 (31.8%) and 49 (24.4%) participants, respectively. Religious considerations and fear of societal judgment were only reported by 5% and 2% of participants, respectively.

A total of 527 desired surgeries were reported, with an average of 2.6 surgeries per participant. The most common surgery desired was tightening of the lower abdomen, (68.7%), followed by medial arm lift (50.7%). The least desired procedures were face and neck lifts.

Group B consisted of 7 participants who underwent BCSs, representing 2.4% of the total sample. Six of the 7 participants were female. In terms of marital status, 5 out of 7 participants were married. All participants in group B reported being unemployed, and all cited dissatisfaction with body image as the primary reason for seeking BCSs. The primary sources of information about BCSs were social media and magazines, as reported by 4 out of 7 participants (57.1%). This was followed by relatives and friends, and finally a specialist surgeon.

**Figure 2.**
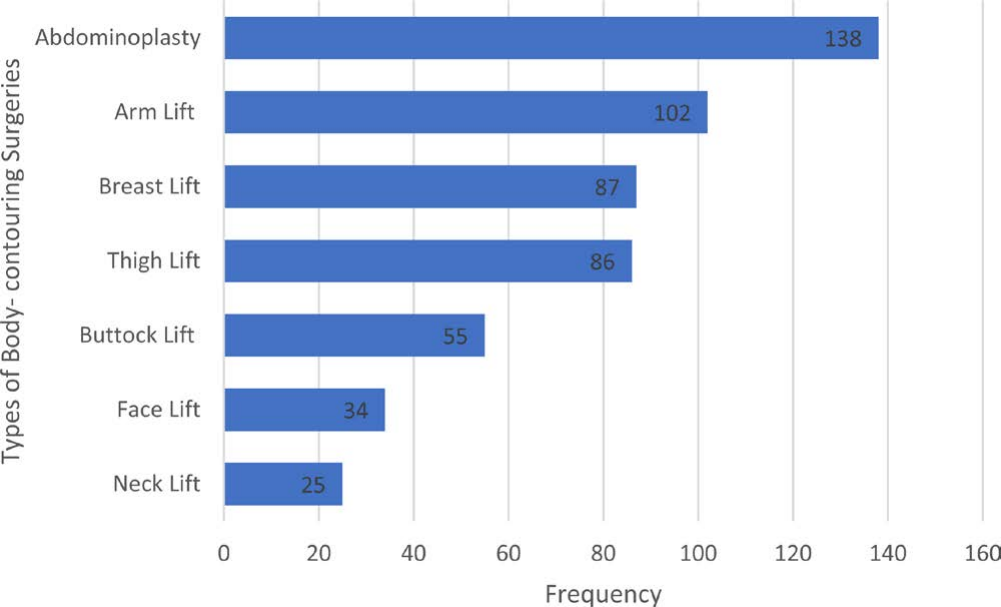
Types of body-contouring surgeries desired by participants in group A.

Abdominoplasty was performed in 4 participants (57.14%), while other types of BCS, such as arm lift, thigh lift, and breast augmentation, were performed in 1 participant (14.3%). Figure 2 illustrates the types of body-contouring surgeries desired by Group A participants.

The most common postoperative complication was seroma formation at the site of abdominoplasty, as reported by 3 out of 7 participants (42.9%). The remaining 4 participants (57.1%) did not experience any complications within 90 days of surgery. There was no statistically significant relationship between the TWL% before BCS and the occurrence of postoperative complications (*P* = .158). A statistically significant relationship was observed between postoperative complications and lower satisfaction levels (*P* = .031).

Most participants who underwent BCSs believed that it improved their performance in daily activities, body image perception, and social relationships. More than half of them (57%) reported a desire to undergo another BCS in the future, and all encouraged others to seek BCSs after weight loss.

## 4. Discussion

The use of bariatric surgery (BS) is increasing rapidly worldwide. Patients often experience significant weight loss and improvements in their quality of life after undergoing BSs.^[2]^ However, they also face new challenges related to body image perception.

Females are more likely to undergo BSs, as our study and other studies showed 50% to 80% of patients were female.^[4,6]^ This high proportion of females undergoing BSs may suggest an increased need to improve social and employment opportunities, as well as address fertility issues compared to males.^[4]^

The prevalence of BS varies across the world. Some studies have reported that Roux-en-Y gastric bypass (RYGB) is performed in more than 55% to 85% of bariatric patients, while others have found that laparoscopic sleeve gastrectomy (LSG) is more prevalent, reaching up to 65% in one study.^[4,7,8]^ This variation may be due to a range of factors, including regional differences, different experiences of surgeons, social trends, and the availability of advanced healthcare systems. However, there was no statistically significant relationship between the type of BS performed and the desire to perform body-contouring surgery (BCS) (*P* = .755), contrary to the findings of Aldaqal et al^[6]^ who reported a higher desire to perform BCSs among patients who underwent RYGB than among those who underwent LSG. To link the desire to perform BCSs to an objective measure in our study, the percentage of total weight loss (TWL%) after BSs was assessed and revealed a statistically significant relationship between TWL% and the desire to undergo BCS (*P* = .025). The TWL% after LSG in this study was 34.3%, compared to 29.0% after RYGB (*P* = .000). More TWL% is reported among our population after LSG specifically, in comparison to literature. In a study by Behrens et al^[9]^ the mean TWL% after LSG was 23.3% in a sample of 52 patients. This difference may be due to the assessment at different points in time from the BS; TWL% was assessed at 21-80 months from the BS in Behrens et al, versus 6 to 55 months in this study, which might reflect potential weight regain over time and indicate the influence of different lifestyles on weight regain.

A large study included more than 900 participants who underwent RYGB in 2008 was conducted by a team from the University of Rochester and reported that BCS prevalence was twice as common among divorced individuals than among married individuals.^[10]^ In our study, the limited number of BCS participants precluded a statistical comparison of these findings. Nonetheless, our analysis found no significant association between marital status and the desire to undergo BCS among Group A (*P* = .734).

In a study conducted in Toronto, Canada, 95.4% of participants expressed a strong desire for BCS, but 88% did not proceed with the procedure because of unaffordable costs. Interestingly, researchers found no significant difference in socio-economic status between participants who eventually underwent BCS and those who did not, indicating the potential effect of subjective considerations when reporting cost as the most common barrier.^[11]^ Accordingly, employment status is commonly thought to be an important factor in a person’s willingness and financial capability to perform a BCS. However, our study found no statistically significant relationship between employment status and the desire to perform BCSs after weight loss (*P* = .319). Employment was not significantly related as well to the degree of knowledge of different BCSs (*P* = .623).

While unaffordable costs are frequently cited as the most significant barrier to BCS in Jordan and globally, the lower prevalence of BCS in Jordan compared to international rates is a cause for concern. This may be attributed to relatively low individual income rates in Jordan and the limited attention given to these issues by insurance companies and healthcare authorities.

Our study found that abdominoplasty was the most desired BCS after weight loss (68.7%), followed by arm lift (50.7%). These results are consistent with the literature, which indicates that abdominoplasty is the most performed BCS procedure following weight loss, accounting for 50% to 60% of all surgeries performed.^[3,5,8]^ Among the 7 participants who underwent BCSs in this study, abdominoplasty was the most frequently performed procedure (57.14%). However, the limited sample size of Group B participants may restrict the generalizability of these findings to the larger population.

Quality of life (QOL) is an important concern for patients both before and after BCSs. Quality of life measures include physical, mental, and social aspects, that can improve independently or in combination. In a study of 30 participants, the Body-Q questionnaire was used to assess the quality of life and satisfaction with body image at 6 and 12 months after BCS. The results showed that performing BCSs significantly improved the general perception of personal appearance, as well as many psychological and social aspects.^[8]^ A systematic review and meta-analysis of 13 QOL studies found that BCSs improved physical, psychological, and social functioning. However, no significant improvements were observed in body image perception, self-esteem, or sexual functioning.^[12]^ In a third large study conducted in Germany, QOL measures, including depression and anxiety levels, were assessed in 300 participants before and after BS and compared with similar measures before and after BCS. Results showed that post-BS participants had fewer symptoms of depression and anxiety, better QOL in all domains, and improved body image on the Multidimensional Body-Self Relations Questionnaire. However, there were no improvements in the observed symptoms after BCSs, despite significant improvements in physical functioning and appearance evaluation. Authors suggested that this could be explained by a ceiling effect, as the differences in observed symptoms pre- and post-BS were already very large, with little room for further improvement.^[13]^ This is consistent with the findings of Song et al,^[14]^ who reported significant and durable improvements in QOL measures based on SF-36 scores at all time points after BS, but not after BCS.

In this study, participants who underwent BCS reported improvements in their performance in daily activities, confidence, self-esteem, and social relationships. We also observed that individuals who experienced postoperative complications expressed less satisfaction with their body image (*P* = .031). This contrasts with previous research which found no association between the occurrence of complications and body image satisfaction.^[8,15]^ It is important to note that our study has potential limitations, including the small sample size of Group B and the lack of a dedicated quality of life (QOL) assessment scale utilized in previous studies.

While social media platforms can offer up-to-date information, they may not be reliable or evidence-based sources of information. Moreover, they do not facilitate effective patient-physician interactions and discussions regarding the potential complications and outcomes of surgical interventions. The prevalence of social media and magazines as the primary sources of information regarding BCSs in our study population is concerning due to the aforementioned drawbacks. Additionally, relying on such sources for information can result in inadequate evaluation of expectations before surgery, potentially affecting patient satisfaction and QOL post-surgery.

## 5. Conclusion

Body contouring surgeries (BCSs) are highly sought after by patients who undergo bariatric surgery and achieve substantial weight loss. Unfortunately, numerous obstacles hinder the pursuit of BCSs, including prohibitively high costs, leading to lower rates of BCSs in our population. By addressing these barriers on a national level and providing comprehensive education on the topic of BCSs, patients can make more informed and health-promoting decisions that will enhance their quality of life.

## Acknowledgments

We would like to express our gratitude to all team members for their hard work and dedication to this research. We also extend our appreciation to Dr Mohammad Khrais and his specialized bariatric surgery center for providing a broad database of participants.

## Author contributions

**Conceptualization:** Marzouq N. Amarin, Amani A. Atallah.

**Data curation:** Izdiad A Atallah.

**Formal analysis:** Izdiad A. Atallah.

**Investigation:** Amani A. Atallah, Majdi M. Khrais, Yazan H. Jaber, Afnan A. Atallah, Omar M. Ismail, Kamel A. Jaber, Taima K. Fkheideh.

**Methodology:** Marzouq N. Amarin, Amani A. Atallah.

**Project administration:** Amani A. Atallah.

**Software:** Izdiad A. Atallah.

**Supervision:** Marzouq N. Amarin, Mohammad Z.A. Rashdan, Raed N. Altaher.

**Writing – original draft:** Amani A. Atallah.

**Writing – review & editing:** Marzouq N. Amarin, Mohammad Z.A. Rashdan, Izdiad A. Atallah, Raed N. Altaher.
